# Supplementary material for: Severe 21st-century ocean acidification in Antarctic Marine Protected Areas
Source: Nat Commun. 2024 Jan 4;15:259. doi: 10.1038/s41467-023-44438-x (PMC10766974; doi:10.1038/s41467-023-44438-x)
Supplement: Supplementary file 1 — Supplementary Information [file 41467_2023_44438_MOESM1_ESM.pdf]

Severe 21st-century ocean acidification in Antarctic Marine Protected Areas

Cara Nissen[1,2*] Nicole S. Lovenduski[1] Cassandra M. Brooks[3]
Mario Hoppema[2] Ralph Timmermann[2] Judith Hauck[2]

[1]Department of Atmospheric and Oceanic Sciences and Institute of Arctic and Alpine Research, University of Colorado Boulder, Boulder, CO, USA.
[2]Alfred Wegener Institut, Helmholtz Zentrum für Polar- und Meeresforschung, Bremerhaven, Germany.
[3]Department of Environmental Studies and Institute of Arctic and Alpine Research, University of Colorado Boulder, Boulder, CO, USA.
[*] Corresponding author: cara.nissen@colorado.edu

This supplementary material contains additional Figures in support of the main manuscript. The following Figures are included in this document:

Fig. S1: Changes in the vertical distribution of Ω_{calc}.

Fig. S2: Temporal evolution of Ω_{calc}.

Fig. S3: Impact of climate change on air-sea CO_2 fluxes.

Fig. S4: Changes in sea-ice cover and ice-shelf basal melt rates.

Fig. S5: Climate-change impacts on pH for the SSP2-4.5 scenario.

Fig. S6: Changes in the vertical distribution of temperature and oxygen.

Fig. S7: Changes in the volume distribution across different Ω_{arag} and Ω_{calc} classes for all continental shelves.

Fig. S8: Grid resolution in the high-latitude Southern Ocean of the global FESOM-REcoM simulations in this study.

Fig. S9: Assessment of model drift: vertical profiles of pH.

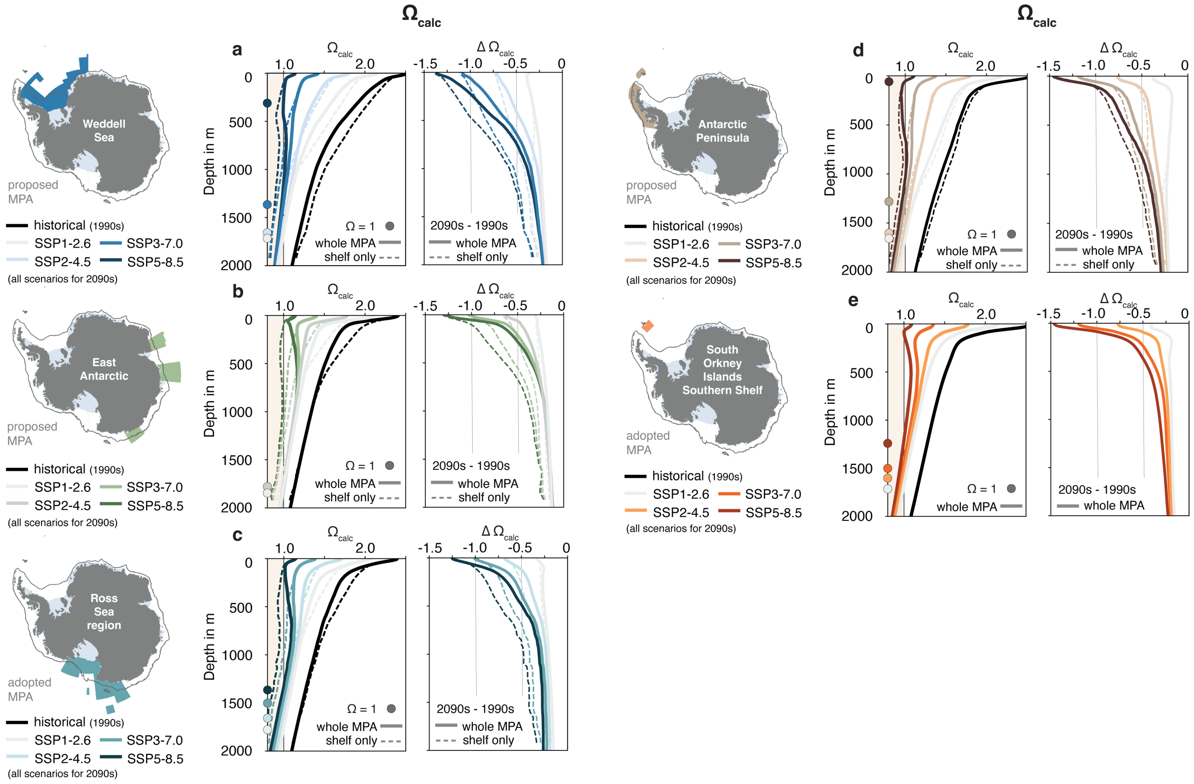

Figure S1: **Changes in the vertical distribution of** Ω_{calc}**.** Vertical profiles of **a** the saturation state with respect to calcite (Ω_{calc}) in the proposed Weddell Sea Marine Protected Area (MPA). Solid lines show the profiles above 2000 m in the whole MPA, and dashed lines show the profile for the continental shelves south of the 2000 m isobath (see dark grey contour in map). The black lines denote the 1990s in the historical simulation, and the colored lines the 2090s in the four emission scenarios. The profiles on the left in each panel show absolute values, and profiles on the right show the change in each property between the 2090s and the 1990s ($\Delta\Omega_{\mathrm{calc}}$). Undersaturated conditions, i.e., Ω_{calc} <1, are highlighted with the colored background, and the depth of Ω_{calc}=1 for the profile of the whole MPA is highlighted with a circle on the y axis. **b**-**e** Same as **a**, but for **b** the proposed East Antarctic MPA, **c** the adopted Ross Sea region MPA, **d** the proposed Antarctic Peninsula MPA, **e** the adopted South Orkney Islands Southern Shelf MPA. Note that only the vertical profiles for the whole MPA are shown in panel **e** (see Methods).

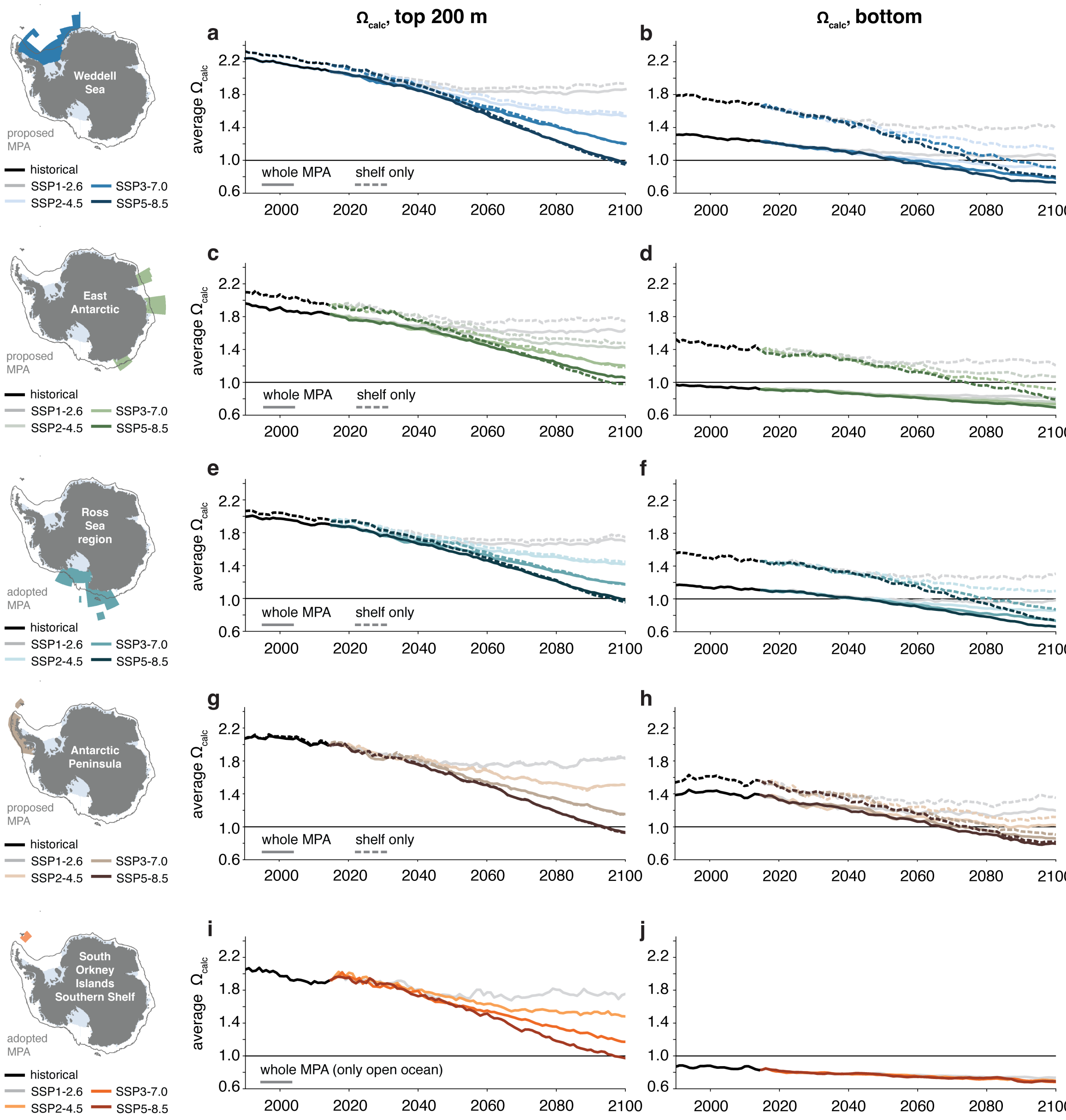

Figure S2: **Temporal evolution of Ω_{calc}.** **a**-**b** Time series of **a** the top 200 m average saturation state with respect to calcite (Ω_{calc}) and **b** bottom Ω_{calc} in the proposed Weddell Sea Marine Protected Area (MPA) for the historical time period (black) and the SSP1-2.6 scenario (light grey), the SSP2-4.5 scenario (light blue), the SSP3-7.0 scenario (intermediate blue) and the SSP5-8.5 scenario (dark blue). Solid lines show the time series for the whole MPA and dashed lines for the continental shelves south of the 2000 m isobath (see dark grey contour in map). **c**-**j** Same as **a**-**b**, but for **c**-**d** the proposed East Antarctic MPA, **e**-**f** the adopted Ross Sea region MPA, **g**-**h** the proposed Antarctic Peninsula MPA, **i**-**j** the adopted South Orkney Islands Southern Shelf MPA. Note that only the time series for the whole MPA are shown in panels **i**-**j** (see Methods).

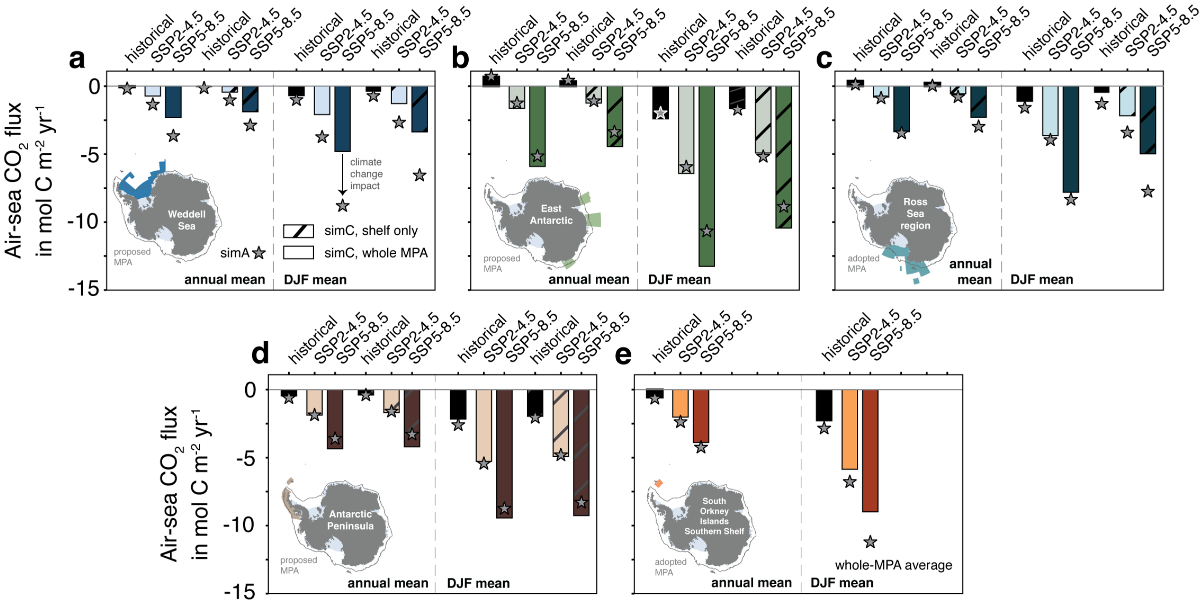

Figure S3: **Impact of climate change on air-sea CO_2 fluxes.** **a** Bars show the air-sea flux of CO_2 in mol C m^{-2} yr^{-1} averaged over the whole proposed Weddell Sea Marine Protected Area (MPA; solid) and on the continental shelves only (hatched) and averaged annually (left bars) and over December-February (DJF; right bars). The black bars denote the flux in the 1990s of the model experiment *simC* (constant climate and increasing atmospheric CO_2, see Methods), and the colors denote the flux in the 2090s of the *simC*-SSP2-4.5 and *simC*-SSP5-8.5 scenario. Negative fluxes denote an oceanic CO_2 uptake. The grey star above each bar shows the corresponding flux in *simA* (varying climate and increasing atmospheric CO_2). The magnitude of the climate-change effect is defined as the difference between the grey star and the respective bar (*simA* minus *simC*, see Methods). **b**-**e** Same as **a**, but for **b** the proposed East Antarctic MPA, **c** the adopted Ross Sea region MPA, **d** the proposed Antarctic Peninsula MPA, and **e** the adopted South Orkney Islands Southern Shelf MPA. Note that only the climate-change effect for the whole MPA is shown in panel **e**.

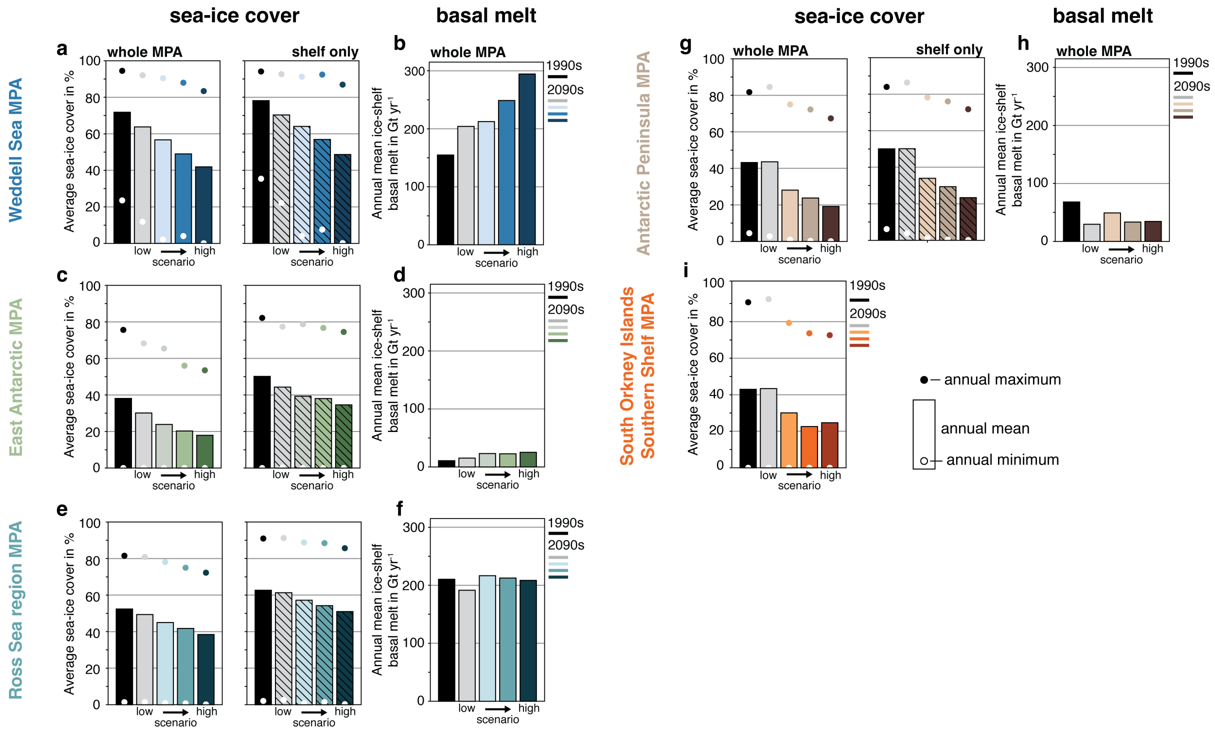

Figure S4: **Changes in sea-ice cover and ice-shelf basal melt rates.** **a**-**b** Average **a** sea-ice cover in % and **b** ice-shelf basal melt rates in Gt yr^{-1} in the Weddell Sea MPA in the 1990s of the historical simulation (black) and the 2090s for the four emission scenarios (colors). Bars denote the annual mean, and white and colored dots in panel **a** show the annual minimum and maximum, respectively. The average sea-ice cover is shown for the whole-MPA average (left) and the continental shelf only (right). **c**-**i** Same as **a**-**b**, but for **c**-**d** the proposed East Antarctic MPA, **e**-**f** the adopted Ross Sea region MPA, **g**-**h** the proposed Antarctic Peninsula MPA, **i** the adopted South Orkney Islands Southern Shelf MPA. Note that only whole-MPA averages are shown in panel i (see Methods).

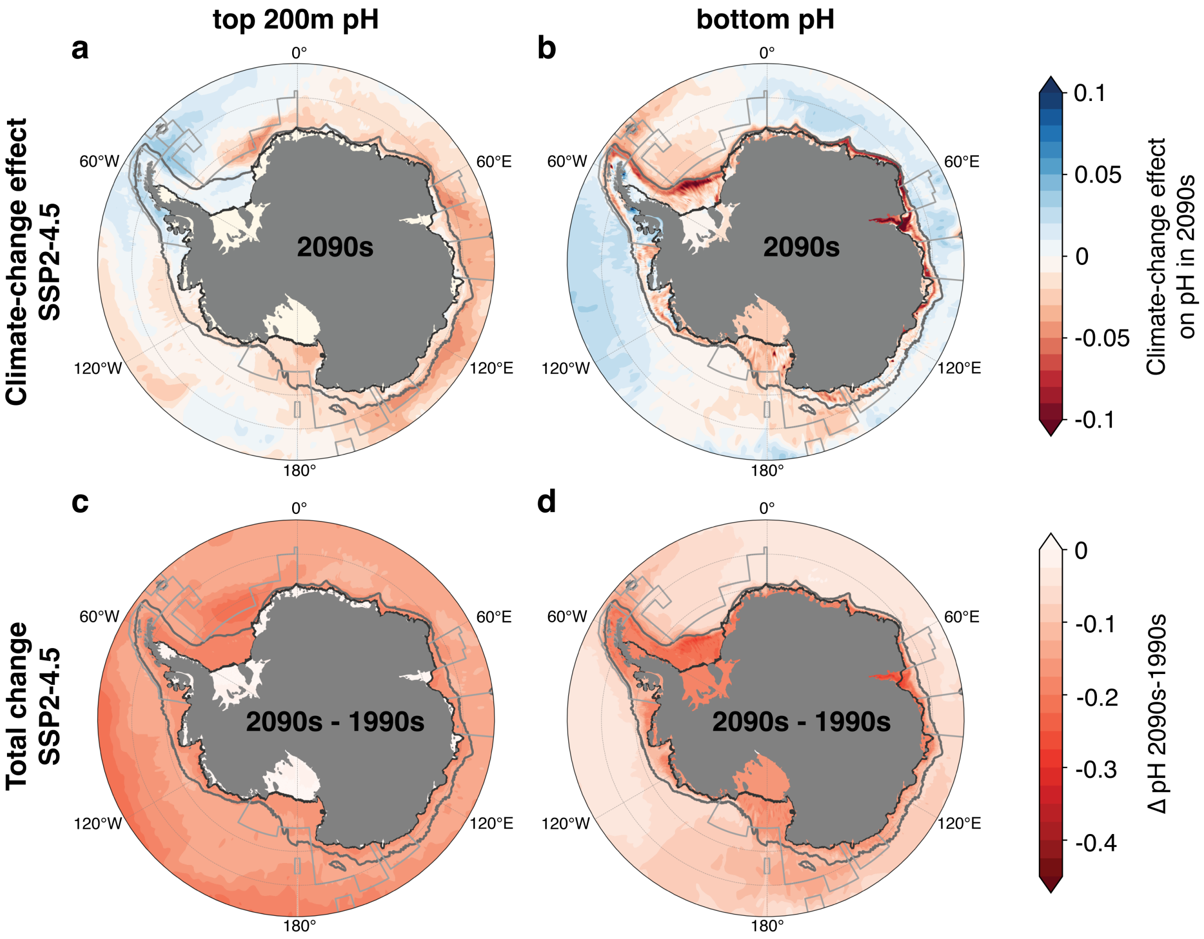

Figure S5: **Climate-change impacts on pH for the SSP2-4.5 scenario.** **a**-**b** Climate-change induced change in pH in the 2090s in the intermediate-emission scenario SSP2-4.5 **a** averaged over the top 200 m of the water column and **b** at the seafloor. The contribution of climate change is calculated from the difference between the simulations *simA* and *simC*, i.e., the simulations with varying and constant climate, respectively (see Methods). **c**-**d** Total change in pH 2090s-1990s in the intermediate-emission scenario SSP2-4.5 **c** averaged over the top 200 m of the water column and **d** at the seafloor. Outlines of the MPAs are shown in light grey, the grey contour denotes the 2000 m isobath, and the ice-shelf front is displayed in black.

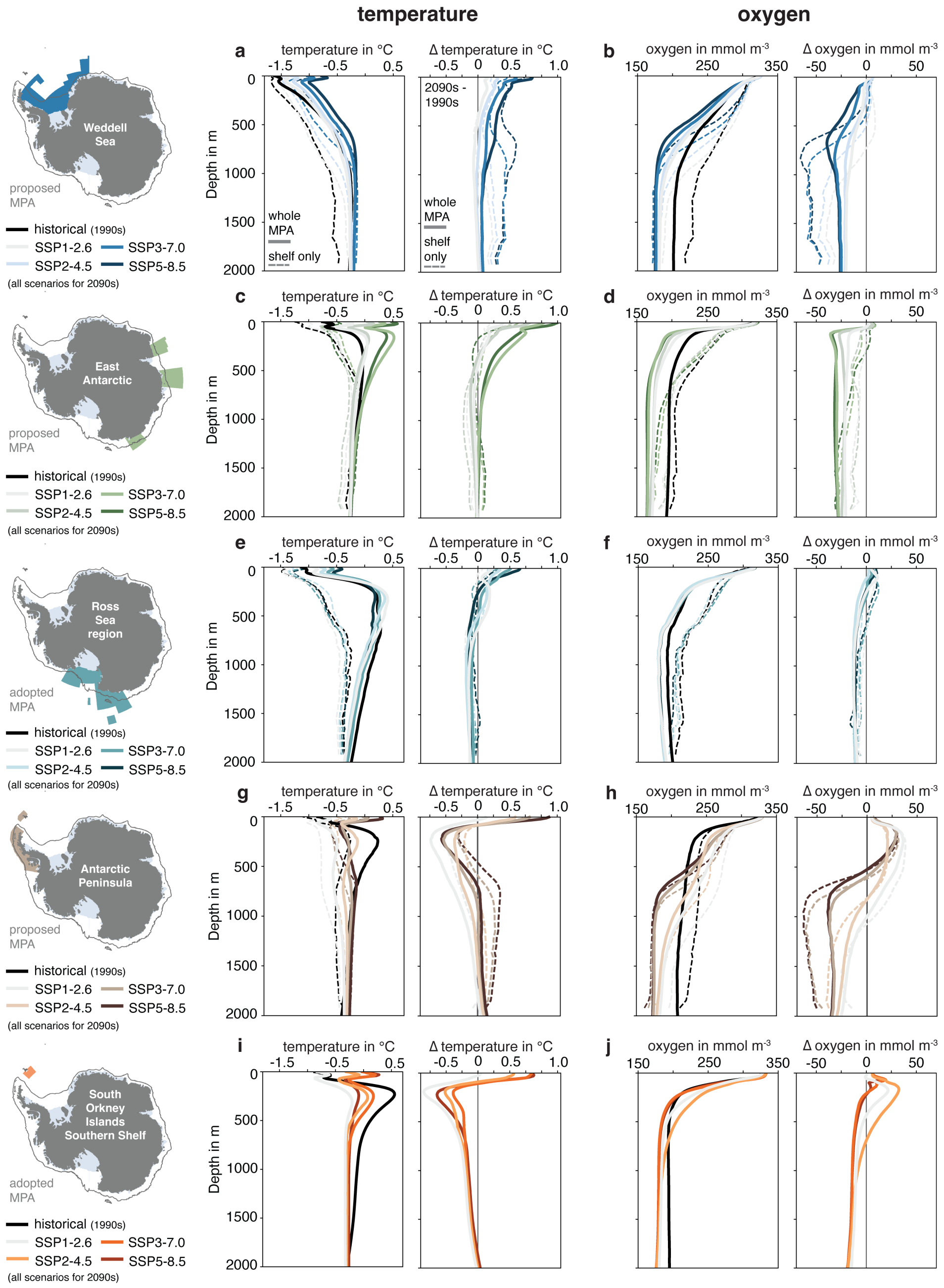

Figure S6: **Changes in the vertical distribution of temperature and oxygen.** Vertical profiles of **a** temperature in °C and **b** oxygen in mmol m^{-3} in the proposed Weddell Sea Marine Protected Area (MPA). Solid lines show the profile above 2000 m in the whole MPA, and dashed lines show the profile for the continental shelves south of the 2000 m isobath (see dark grey contour in map). The black lines denote the 1990s in the historical simulation, and the colored lines the 2090s in the four emission scenarios. The profiles on the left in each panel show absolute values, and profiles on the right show the change in each property between the 2090s and the 1990s. **c**-**j** Same as **a**-**b**, but for **c**-**d** the proposed East Antarctic MPA, **e**-**f** the adopted Ross Sea region MPA, **g**-**h** the proposed Antarctic Peninsula MPA, **i**-**j** the adopted South Orkney Islands Southern Shelf MPA. Note that only the vertical profiles for the whole MPA are shown in panels **i**-**j**.

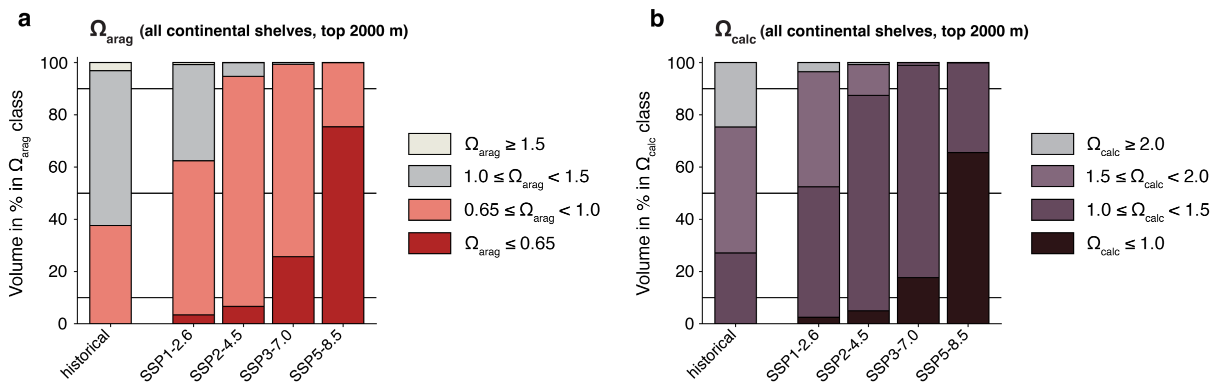

Figure S7: **Changes in the volume distribution across different Ω_{arag} and Ω_{calc} classes for all continental shelves. a** Distribution of waters across different classes of the saturation state with respect to aragonite (Ω_{arag}) on all continental shelves south of the 2000 m isobath (see dark grey contour in e.g., Supplementary Fig. 5). Results are shown for the 1990s of the historical simulation and for the 2090s in the four emission scenarios. **b** Same as **a**, but for the distribution of waters across different classes of the saturation state with respect to calcite (Ω_{calc}). Note the different classes of saturation states shown for aragonite and calcite.

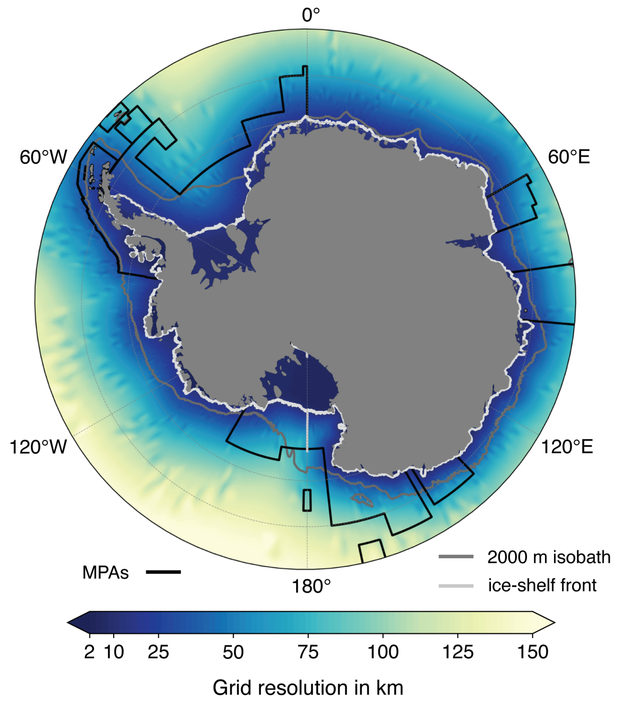

Figure S8: **Grid resolution in the high-latitude Southern Ocean of the global FESOM-REcoM simulations in this study.** Black outlines denote the Marine Protected Areas. The dark grey contour shows the 2000 m isobath; the light grey contour indicates the ice-shelf front.

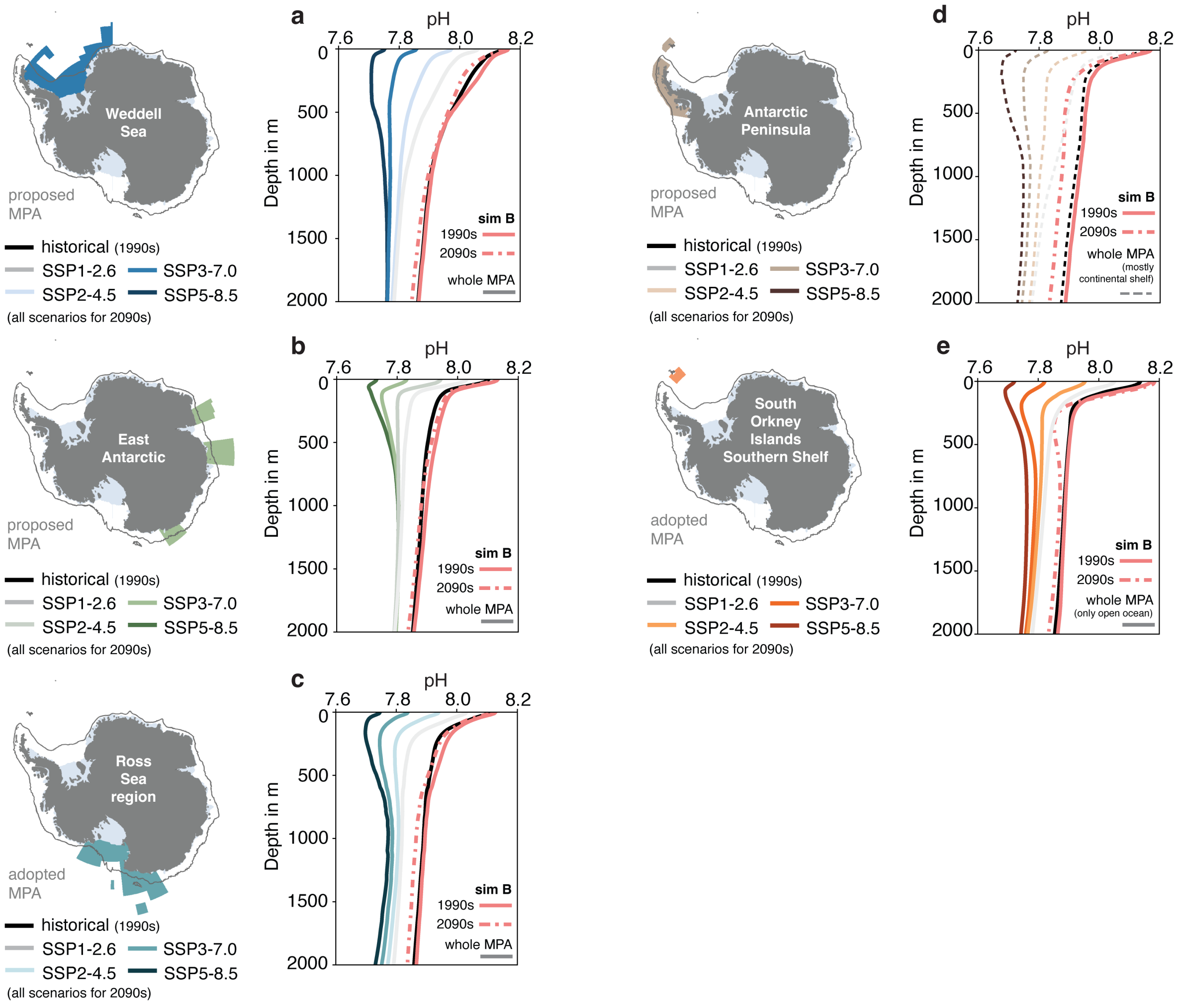

Figure S9: **Assessment of model drift: vertical profiles of pH.** Vertical profiles of pH in **a** the proposed Weddell Sea Marine Protected Area (MPA). The pH in the control simulation *simB* is shown in pink for the 1990s (solid) and the 2090s (dashed dotted). All other lines are the same as the profiles for the whole MPA in Fig. 3 of the main text and denote the average profile in *simA* in the 1990s (black) and the 2090s for the four emission scenarios (colors). **b**-**e** Same as **a**, but for **b** the proposed East Antarctic MPA, **c** the adopted Ross Sea region MPA, **d** the proposed Antarctic Peninsula MPA, **e** the adopted South Orkney Islands Southern Shelf MPA.
